# Optimization of the Solid-state Fermentation and Properties of a Polysaccharide from *Paecilomyces cicadae* (Miquel) Samson and Its Antioxidant Activities *In Vitro*

**DOI:** 10.1371/journal.pone.0087578

**Published:** 2014-02-03

**Authors:** Xueyong Ren, Liang He, Junwen Cheng, Jianmin Chang

**Affiliations:** 1 College of Materials Science and Technology, Beijing Forestry University, Beijing, P.R. China; 2 Key Laboratory of Biological and Chemical Utilization of Zhejiang Forest Resources, Institute of Biological Technology, Zhejiang Forestry Academy, Hangzhou, P.R. China; Taipei Medical University, Taiwan

## Abstract

The culture conditions for the yield of a polysaccharide (PCPS) produced by *Paecilomyces cicadae* (Miquel) Samson on solid-state fermentation were investigated using response surface methodology (RSM). Plackett–Burman design (PBD) was applied to screen out significant factors, followed by the paths of steepest ascent to move to the nearest region of maximum response. Then Box-Behnken design (BBD) was conducted to optimize the final levels of the culture conditions. After analyzing the regression equation and the response surface contour plots, relative humidity 56.07%, inoculum 13.51 mL/100 g and temperature 27.09°C were found to be the optimal key parameters for PCPS production. The maximum predicted yield of PCPS was 10.76 mg/g under the optimized conditions. The resulting PCPS (FPCPS) generated at optimal conditions was purified by chromatography column and found to be composed of mannose (43.2%), rhamnose (32.1%), xylose (14.5%) and arabinose (10.2%). Based on the size exclusion chromatography combined with multi-angle laser light scattering (SEC-MALLS) analysis, FPCPS adopted a Gaussian coil conformation in 0.1 M NaNO_3_ solution with 3.75×10^6^ g/mol of the weight-average molar mass (M_w_) and 41.1 nm of the root-mean square radius (Rg^2^)_z_^1/2^. Furthermore, both of the polysaccharides were revealed to have strong antioxidant activities by evaluating in DPPH radical, superoxide radicals and hydroxyl radical assay. These data suggest the polysaccharides of *Paecilomyces cicadae* (Miquel) Samson produced by solid-state fermentation could be explored as potential natural antioxidants.

## Introduction

Medicinal fungi can secrete sorts of important secondary metabolic products, which have a wide range of applications in pharmaceutical and food industries. Among them, polysaccharides have been well studied due to their novel functionality, constant reproduction and stable cost [1]. They can be incorporated in food industry as thickeners and bio-emulsifiers to improve food quality and texture [2]. In the pharmaceutical industry, they were utilized as dietary free radical scavenger for the prevention of oxidative damage [3], anti-inflammatory drug for health protection [4], as anti-HIV agent [5] to enhance immune system.

Polysaccharides with different structures have been found to exist as various chain conformations in solution [6]. The molecular weight and chain conformation of the polysaccharides significantly affected their bioactivities [7]. In authors' point of view, some information on the molecular characteristics of the polymer molecules such as Z-averaged root-mean square radius of gyration (Rg^2^)_z_^1/2^, weight-average molecular weight, polydispersity index and solubility in dilute solutions could provide insights into the physico-chemical behavior and indicate effective application of the biopolymer [8]. Therefore, it is essential to acquire basic parameters of the biomacromolecules for the successful interpretation of their bioactivities mechanism.

*Paecilomyces cicadae* (Miquel) Samson ( = *Isaria sinclairii*), as the anamorph stage of *Cordyceps cicadae*, is an entomogenous and medicinal fungus, which has attracted considerable attention because the extractives from its mycelium or culture broth have been reported to present multiple therapeutic activities [9]. Our previous work showed that the exopolysaccharide from the submerged fermentation was uncovered to have strong reductive power and potent inhibiting power for hydroxyl radical [10]. The isolated polysaccharides from *P. cicadae* was reported to show the activation of macrophages through the TLR4 signaling pathway, increase of in interferon IFN-*γ* production by Peyer's patch cells and immunomodulatory function by RAW 264.7 cells [11]–[12]. So far, potential industrial market demands that more intensive research and development should be undertaken on this genus. However, rare natural resources limit the development of *Paecilomyces* with a specific host and a strictly conditioned environment to grow. Hence the requirements for culturing the anamorph of *Paecilomyces* become more beneficial to address the issue of exploitation commercially.

Mycelial growth and the accumulation of polysaccharide produced by *P. cicadae* in fermentation are strongly influenced by fermentation conditions. Thus, the investigation of batch cultivation conditions is a shortcut for utilizing *P. cicadae* to improve polysaccharide production. As mentioned above, the polysaccharides obtained from fruiting body of *P. cicadae* or liquid-state fermentation has been well exploited. However, the factors on solid-state fermentation of *P. cicadae* have not been reported, nor any references made of the conformation and their biological activities. Statistical screening methodology is a powerful and useful tool in searching the key factors rapidly from a multivariable system [13]. Herein, a Plackett-Burman statistical design was applied to screen out the significant factors, followed by the paths of steepest ascent to move to the nearest region of maximum response [14]. Then three main factors were chosen in the present study for further optimization using response surface methodology (RSM), employing a three-level and three-variable Box-Behnken design [15]. The main aim of this work was to optimize the solid-state fermentation conditions of *P. cicadae* based on the statistical analysis and to elucidate the characterization of highly purified soluble polysaccharide and evaluate its antioxidant activities, which will provide the foundation for future pharmacological and biochemical studies.

## Materials and Methods

### Strain and solid-state fermentation

ZJ001, a strain of *P. cicadae* used in this study was originally isolated from *Cordyceps cicadae* collected from a forestry center located in Wuchao Mountain, Hangzhou, Zhejiang Province, China. The authorization of this fungus study was issued by Zhejiang Forestry Academy, China. It was maintained on potato dextrose agar (PDA) supplemented with 10 g/L wheat bran and 10 g/L silkworm pupa powder at 4°C. All batch experiments were carried out in Erlenmeyer flasks (250 mL) with different composition of fermentation medium according to the design as follows. The soybean residue was kindly provided by an agro-industry plant located in the northeast region of Heilongjiang Province, then dried to 2% humidity in an oven with air circulation and forced renewal at 70°C for 24 h and ground in a mill with the particle size of approximately 2 mm. The flasks, after autoclaving at 121°C for 25 min and cooling to 25°C, were inoculated with mycelia from 10-days-old plates. The mixture was shaken at the seventh day to help the aeration and homogenization of substrate. After incubation, the fungal biomass was oven-dried at 50°C for 12 h and used for the determination of polysaccharides.

### Estimation of the yield of PCPS

After cultivation, the fungal biomass was ground in a sample mill to pass through No. 60 mesh after oven drying for 3 days at 60°C. The powdered material was refluxed in 80% ethanol for 6 h to remove some colored materials, monosaccharides, oligosaccharides, and small molecule materials. Then the cooled extract was discarded and the residue was washed with 95% ethanol, anhydrous ethyl alcohol, acetone and diethyl ether respectively. The residue was dried at room temperature for 24 h prior to extraction. Subsequently, the residue was blended with distilled water at 80°C for 2 h in a water bath and the residue was re-extracted under the same condition. The combined extract obtained from the cultivation medium by filtration through Whatman No.1 filter paper was concentrated to minimum volume under diminished pressure at 50°C and then precipitated with 4 volumes of 95% alcohol at 4°C overnight and the precipitation was centrifuged at 4000 rpm for 15 min and the supernatant was removed. The sediment was dissolved in distilled water to certain volume in which the PCPS concentration was determined according to the classical method of Dubois et al. using glucose solution as a standard reference [16].

### One factor at a time

In each experiment, one factor was changed with the other factors remaining constant. Different carbon sources (glucose, dextrin), nitrogen sources (peptone), mineral sources (CaCl_2_), inoculum, initial pH, the concentration of media components and relative humidity were initially studied by single factor experiments. Although single variable method is time-consuming, and it overlooks the interaction between different factors, this method is propitious to the selection of level in PBD, making the result more reasonable and credible.

### Plackett–Burman design for screening

Plackett–Burman design (PBD), a powerful and useful tool in rapidly searching for the key factors from a multivariable system, has been used for screening the key factors prior to optimization [17].

Each independent variable was tested at two levels, high and low, which are denoted by (+) and (−), respectively. The experimental design with the name, symbol code, and actual level of the variables is shown in Table 1, whereas Table 2 shows the detail of the design matrix.

**Table 1:** Experimental field definition for the Plackett–Burman design.

Symbol code	Factors	Experimental values	
		Low level (−1)	High level (+1)
A (g/L)	Glucose	5	10
B (g/L)	Peptone	2	8
C (g/L)	CaCl_2_	0.5	2
D (g/L)	Dextrin	5	15
E(%)	Relative humidity	40	70
F	Initail pH	4	8
G (mL/100 g)	Inoculum	4	20
H (°C)	Temperature	20	40
I–K	Dummy factors	-	-

**Table 2:** Plackett–Burman design matrix with response value.

Run	Variable levels											Yield of PCPS (mg/g)
	A	B	C	D	E	F	G	H	I	J	K	
1	1	1	−1	−1	−1	1	−1	1	1	−1	1	8.45±0.013
2	−1	1	1	−1	1	1	1	−1	−1	−1	1	5.85±0.027
3	1	−1	1	1	1	−1	−1	−1	1	−1	1	6.65±0.004
4	1	−1	1	1	−1	1	1	1	−1	−1	−1	8.75±0.016
5	−1	−1	−1	−1	−1	−1	−1	−1	−1	−1	−1	4.25±0.028
6	−1	−1	1	−1	1	1	−1	1	1	1	−1	9.80±0.009
7	1	1	−1	1	1	1	−1	−1	−1	1	−1	7.30±0.011
8	−1	1	1	1	−1	−1	−1	1	−1	1	1	8.50±0.017
9	−1	1	−1	1	1	−1	1	1	1	−1	−1	9.35±0.005
10	1	1	1	−1	−1	−1	1	−1	1	1	−1	4.40±0.008
11	1	−1	−1	−1	1	−1	1	1	−1	1	1	8.35±0.027
12	−1	−1	−1	1	−1	1	1	−1	1	1	1	4.15±0.014

In this work, eight factors and their levels were chosen based on the results of one-factor-at-a-time, which are essential for the mycelial growth and PCPS production of *P. cicadae* [18]. Three dummy variables were studied in 12 experiments to calculate the standard error, and PBD was based on the first order polynomial model:
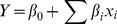
(1)


Where *Y* is the response (PCPS), *β_0_* is the model intercept and *β_i_* is the linear coefficient, and *x_i_* is the level of the independent variable. From the regression analysis of the variables, the significant levels at 95% level (*p*≤0.05) were considered to have greater impact on PCPS production.

### Path of the steepest ascent experiment

After the screening design identifying the significant variables, the steepest ascent was employed to move the experimental region of the response in the direction of the optimum, by appropriately changing the range of the selected variables. The path starts from the design center of the factorial design (the screening design) and ends when no further improvement in the response can be achieved. While a maximum value is found, that point could be applied as the center point for the following optimization experimental design [19].

### Response surface methodology (RSM)

Response surface methodology is a collection of statistical tools and techniques for constructing and exploring an approximate functional relationship between a response variable and a set of design variables. It is possible to derive an expression for the performance measurement based on the responded values obtained from experiments at some particular combination of the input variables [20].
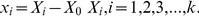
(2)


Where *x_i_* is the dimensionless value of an independent variable; *X_i_*, the real value of an independent variable; *X_0_*, the real value of an independent variable at the central point and *X_i_* is the step change.

The experimental design was a Box–Behnken design experimental plan with three cultivation conditions, i.e., temperature, relative humidity and inoculum. In this study, the experimental plan consisted of 17 trials and the value of the dependent response was the mean of two replications. The second-order polynomial coefficients were calculated and analyzed using the ‘Design Expert’ software (Version 8.0.5b, State-Ease Inc., Minneapolis, USA) statistical package. Statistical analysis of the model was performed to evaluate the analysis of variance (ANOVA).

### Fractionation of PCPS

The crude PCPS was dissolved in distilled water with ultrasonic treatment and then deproteined using Sevag method. Briefly, the crude PCPS was mixed with Sevag reagent (chloroform: n-butanol at 4∶1 v/v) at a ratio of 3∶1 (v/v). The mixture was allowed to react at room temperature for 40 min. After centrifugation for 10 min at a speed of 8000 rpm, the supernatant was precipitated again with 4 volumes of 95% alcohol to produce sediment. And then, the deproteined PCPS (100 mg) was dissolved in distilled water and filtered with membrane (0.45 µm, Millipore) before it was loaded in a diethylaminoethyl (DEAE)-52 column (2.0 cm×35 cm). After the column was equilibrated with distilled water, it was washed with a range of 0.05 to 1.5 M NaCl at a flow rate of 1.0 mL/min, with 3 mL fraction collected. The major peak was pooled, dialyzed completely and finally lyophilized. The obtained polysaccharide was re-dissolved in 0.1 M NaCl and subjected to a Sephadex G-200 column (1.5 cm×60 cm), which was eluted by a 0.1 mol/L NaCl at a flow rate of 0.5 mL/min. Finally, the fraction was desalted thoroughly and lyophilized to a cotton-like polysaccharide (FPCPS).

### Chemical and physical analysis of FPCPS

The total sugar content of FPCPS was evaluated by Dubois method [16]. The standard curve was made using a series of concentration of glucose to determine the absorbance of 620 nm. The protein content of samples was measured by the Lowry method using bovine serum albumin (BSA) as a standard [21]. The monosaccharide components of FPCPS were analyzed by reverse-phase HPLC according to PMP (1-phenyl-3-methyl-5-pyrazolone) derivatization procedures with slight modification [22].

The major structural information of FPCPS was analyzed using a Fourier-transform infrared spectrophotometer (Nexus IS10 FTIR, Thermo Nicolet, USA). FPCPS sample was pressed into KBr pellet at a sample to KBr weight ratio of 1∶20. The FTIR spectra were recorded in the range of 4000–400 cm^−1^.

Molecular weight of FPCPS was measured by size exclusion chromatography with a multi-angle laser light scattering system (SEC-MALLS). The system included a pump (S-1500, SSI, USA), a degasser (GASTORR TG-14, GenTech Scientific Inc., USA), an injection valve (High-Pressure Injection system, Wyatt Technology, USA) fitted with a 100 µL loop, SEC columns (TSK G3000 PW_XL_, TOSOH, Japan), a multi-angle laser light scattering detector (DAWN HELEOS II, Wyatt Technology, USA)(λ_0_ = 658 nm), and a refractive index detector (RID-10A, SHIMADIU corporation, JAPAN). Samples were dissolved directly in ultrapure water (1–3 mg/mL), and filtered through 0.22 µm filter membranes (Millipore) prior to injection into the SEC/MALLS system. Nitrate buffer was used as the mobile phase, which containing 0.1 M NaNO_3_ and 0.02% NaN_3_, then filtered over a filter membrane with pore size 0.22 µm, and degassed by ultrasonic cleaner for several minutes [23].

### Antioxidant activities of PCPS

The free-radical scavenging capacity of water extract, PCPS and FPCPS were measured by 1, 1-diphenyl-2-picryldydrazyl (DPPH) test according to the method of Blois [24] with minor modifications.

The superoxide radical assay was measured by the method of Robak and Gryglewski [25] with some modifications. The samples were dissolved in distilled water at 0 (control), 0.05, 0.1, 0.2, 0.5, 1.0 or 1.5 mg/mL. Before the generation of superoxide radical, a 0.1 mL of sample solution was added with 1.0 mL of 16 mM Tris-HCl buffer (pH8.0) and 1.0 mL of that containing 0.072 mM NBT. The reaction of this experiment was started by injecting 1.0 mL 16 mM Tris-HCl buffer (pH8.0) containing 0.03 mM PMS. After incubation at 25°C for 5 min, the absorbance at 560 nm was measured. The superoxide radical effect was calculated using the following Eq. (3). Herein, ascorbic acid was used as a positive control standard.
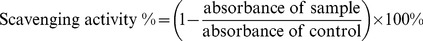
(3)


The hydroxyl radical system generated by the Fenton reaction was evaluated *in vitro* according to the method described by He et al. [26]. Briefly, samples of various concentrations (0–1.0 mg/mL) were mixed with 1.0 mL of brilliant green (0.435 mM), 0.5 mL FeSO_4_ and 1.5 mL of 3.0% H_2_O_2_. And then the solution was kept for 20 min at room temperature and the absorbance at 624 nm was recorded. The scavenging activity of the hydroxyl radical was calculated as follows (Eq.4).
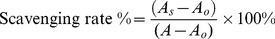
(4)


Where *A_s_* is the absorbance in the presence of the sample, and *A_o_* is the absorbance of the control in the absence of the sample, and *A* is the absorbance without the sample and Fenton reaction system.

## Results and Discussion

### Screening the factors affecting PCPS production in PBD

Eight variables were chosen in the PBD fermentation process to efficiently screen out the key factors on the PCPS production (in Table 1). The data reported in Table 2 showed a substantial variation in PCPS yield among the 12 experimental runs, going from 4.15 mg/g to 9.80 mg/g under different levels of factors, suggesting that the screened parameters were important for the solid-state fermentation of the biomass and PCPS.

From the regression analysis of PBD in Table 3, the fitting model for PCPS production was highly significant (*p* = 0.0044). A ratio of adequate precision (19.12) confirmed that the model was able to adequately navigate the design space. The goodness of the model was checked by the determination coefficient R^2^ (0.9923), explaining 99.23% of the variability of the response. Among those factors, relative humidity, inoculum and temperature showed the significant effects on the response (*p*<0.05). In particular, relative humidity and temperature offered a positive effect on PCPS production, while inoculum seemed to have a negative effect. Instead, dextrin, peptone and initial pH as well as other variables presented little or no influence within the considered range (*p*>0.05). The variables with insignificant effect were not included in the next optimizing step.

**Table 3:** Analysis of variance for Plackett–Burman factorial model.

Variable		Level			Relative Significance	
Code	Term	Low (−1)	High (+1)	Effect	*t*-test	*p*-value
A	Glucose (g/L)	5	10	0.067	1.69	0.1930
B	Peptone (g/L)	2	8	0.063	1.58	0.2103
C	CaCl_2_ (g/L)	0.5	2	0.070	1.76	0.1773
D	Dextrin (g/L)	5	15	0.12	3.02	0.0572
E	Relative humidity (%)	40	70	0.29	7.30	0.0052*
F	Initail pH	4	8	0.093	2.34	0.1011
G	Inoculum (mL/100 g)	5	20	−0.14	3.52	0.0416*
H	Temperature (°C)	20	40	0.69	17.38	0.0004*

Adequate precision = 19.122, Press = 5.72, R^2^ = 0.9923, adj-R^2^ = 0.9717.

* Identified variables with a significant effect on the response (*p*-value<0.05).

### Steepest ascent

Based on the regression analysis of the screening design, the path of steepest ascent was then applied to find the most suitable direction for changing the variable ranges. On the basis of Eq. (1) and Table 3, the direction of steepest ascent should increase concentration of relative humidity (E) and temperature (H), decrease concentration of inoculum (G) in order to approach the optimal experimental region of maximum response. Five sets of experimental design of the steepest ascent and corresponding results were given in Table 4. The yield of PCPS peaked at the third step and no further improvement could be achieved in the response when E, G and H was selected to be 55%, 12 mL/100 g, and 28°C, which suggested that it was near the region of maximum response (PCPS). Accordingly, these levels of the three factors in Run 3 were set as the center point of BBD.

**Table 4:** Steepest ascent experiments to move the experimental region towards the maximum yield of PCPS.

Run	Variable levels			Yield of PCPS (mg/g)
	Relative humidity (%)	Inoculum (mL/100 g)	Temperature (°C)	
1	45	20	20	4.85
2	50	16	24	6.4
3	55	12	28	9.3
4	60	8	32	7.95
5	65	4	36	5.15

### Box-Behnken optimization of the yield of PCPS

Preliminary trials enabled the range of relative humidity from 50 to 60%, inoculum (6–18 mL/100 g) and temperature (24–32°C) to be fixed. In the present step, experiments were planned to obtain a quadratic model consisting of 12 trials plus 5 central points. The design matrix of the variables in coded units is given in Table 5 along with the predicted and experimental values of response. Each run was performed in duplicate and thus the values of PCPS were the average of two sets of experiments, while the predicted values of responses were obtained from quadratic model fitting techniques using the software mentioned above.

**Table 5:** Box–Behnken design matrix along with the experimental and predicted values of the yield of PCPS.

Run order	Relative humidity (x_1_)		Inoculum (x_2_)		Temperature (x_3_)		The yield of PCPS (g/L)	
	Coded level	Real level (%)	Coded level	Real level (mL/100 g)	Coded level	Real level (°C)	Experimental	Predicted
1	−1	50	1	18	0	28	7.65±0.008	7.09
2	1	60	0	12	−1	24	8.05±0.015	7.79
3	0	55	0	12	0	28	10.5±0.022	10.5
4	1	60	−1	6	0	28	6.75±0.026	7.31
5	−1	50	−1	6	0	28	5.65±0.003	5.56
6	0	55	0	12	0	28	10.05±0.007	10.5
7	0	55	−1	6	1	32	6.55±0.018	6.48
8	0	55	1	18	−1	24	9.25±0.004	9.33
9	0	55	0	12	0	28	10.25±0.028	10.5
10	−1	50	0	12	1	32	4.45±0.031	4.71
11	0	55	0	12	0	28	10.7±0.016	10.5
12	1	60	1	18	0	28	8.25±0.006	8.44
13	0	55	1	18	1	32	4.50±0.012	4.8
14	1	60	0	12	1	32	8.20±0.009	7.71
15	−1	50	0	12	−1	24	7.10±0.015	7.59
16	0	55	−1	6	−1	24	5.20±0.013	4.9
17	0	55	0	12	0	28	11.00±0.005	10.5

By applying multiple regression analysis on the experimental data given in Table 5, the second-order polynomial equation for the yield of PCPS is presented as follows:
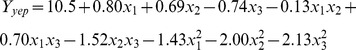
(5)where *Y_yep_* (mg/g) is the predicted response of the yield of PCPS.

The statistical significance of the second-order model (3) and all the coefficient estimates were checked by analysis of variance (ANOVA) and data shown in Table 6. It is fairly clear that the quadratic regression model is highly significant, as is evident from the *F*-test with a very low probability value. The value of adj-R^2^ (0.9380) suggests that the total variation of 93.8% for the yield of PCPS is attributed to the independent variables. The value of R (0.9864) shows a close agreement between the experimental results and the theoretical values predicted by the polynomial model [27]. It also can be seen that all regression coefficients are significant except for two linear terms (relative humidity, inoculum).

**Table 6:** Results of regression analysis of a full second-order polynomial model for optimization of the yield of PCPS.

Model term	Coefficient estimated	S.E.	Sum of Squares	*F*-Value	Probability (*p*)>*F*
Intercept	10.5	0.24	2.96		
x_1_ (Relative humidity)	0.80	0.19	5.12	17.38	0.0042
x_2_ (Inoculum)	0.69	0.19	3.78	12.83	0.0089
x_3_(Temperature)	−0.74	0.19	4.35	14.77	0.0064
x_1_x_2_	−0.13	0.27	0.063	0.21	0.6591
x_1_x_3_	0.70	0.27	1.96	6.65	0.0365
x_2_x_3_	−1.52	0.27	9.30	31.57	0.0008
x_1_^2^	−1.43	0.26	8.55	29.02	0.0010
x_2_^2^	−2.00	0.26	16.84	57.16	0.0001
x_3_^2^	−2.13	0.26	19.01	64.53	<0.0001
Residual	0.083	7	2.06		
Lack-of-fit	0.06	3	1.51	3.62	0.1229
Pure error	0.022	4	0.55		
Cor total	76.07	16			

R^2^ = 0.9729, adj-R^2^ = 0.9380, R = 0.9864 and Adequate precision = 13.902.

**Figure 1:**
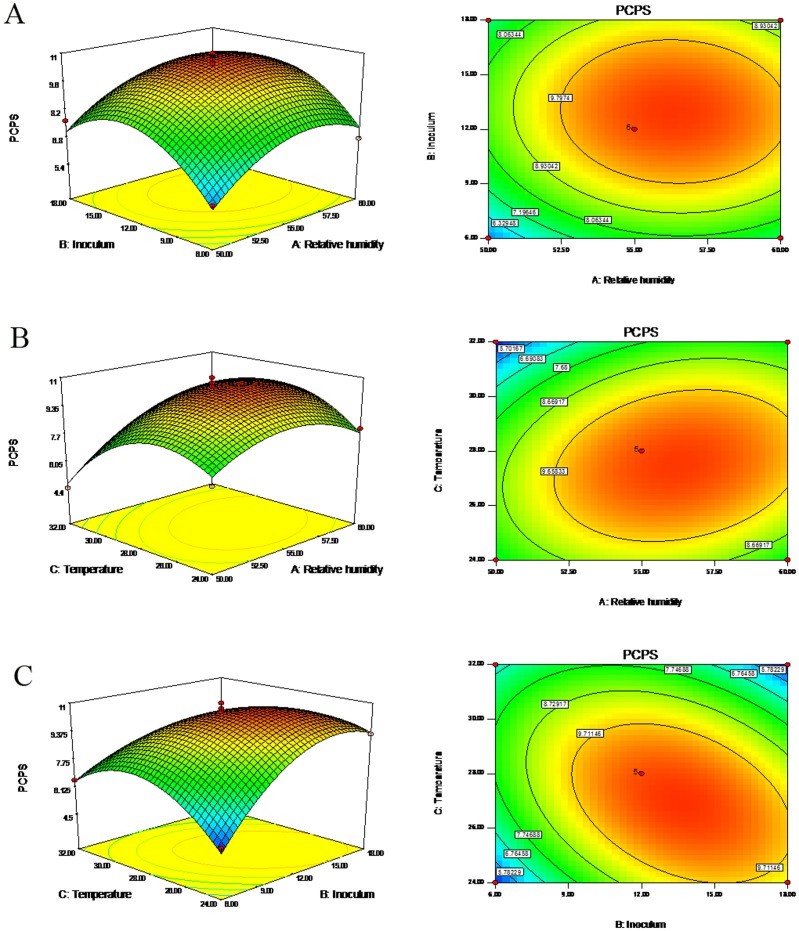
Response surface (left) and contour (right) plots for the effects of three variables on PCPS from *P. cicadae*. (A), response surface plot showing the mutual effect of relative humidity and inoculum on the production of PCPS; (B), response surface plot showing the mutual effect of relative humidity and temperature on the production of PCPS; (C), response surface plot showing the mutual effect of inoculum and temperature on the production of PCPS.

The 3D-surface plot and 2D-projection could visually show the response over a region of interesting factor levels, the relationship between the response and experimental levels of each variable. Figure 1 was the fitted response surface plots and their corresponding contour plots for the yield of PCPS generated by the predicted model, respectively. It was easily observed from Figure 1A that the yield of PCPS significantly increased upon relative humidity up to about 56.07%, but decreased a little beyond this point, reaching a maximum yield of 10.7 mg/g. The effect of inoculum on the yield was also sensitive within the tested range, which could be proved by the *p*-value (0.0064) in Table 6. And the significant interaction of relative humidity and temperature could be easily explained by its elliptical shape of the contour plot and *p*-value (0.0365<0.05). According to the response curves in Figure 1B, the yield of PCPS resulted in a linear increase in degree of temperature, and then reduced slightly with the designed range of relative humidity from 56% to 60%. It was also noticed in Figure 1C that the response presented downward movement when the value of inoculums was higher than 13.51 mL/100 g, indicating the existence of the maximum predicted value of PCPS yield.

By solving the inverse matrix from Eq. (5), the optimum values of the test variables in uncoded units were relative humidity 56.07%, inoculum 13.51 mL/100 g and temperature 27.09°C. Under the optimal conditions, the maximum predicted yield of PCPS was 10.76 mg/g of fermentation liquor from *P. cicadae* in submerged fermentation. In order to verify the prediction of the model, the optimal reaction conditions were applied to three independent replicates to generate PCPS synthesis. The average yield of PCPS was 10.71±0.156 mg/g (N = 3). This demonstrated the validation of the developed RSM model. The good correlation between these results confirmed that the response model was adequate to reflect the expected optimization.

### Properties of PCPS from P. cicadae

**Figure 2:**
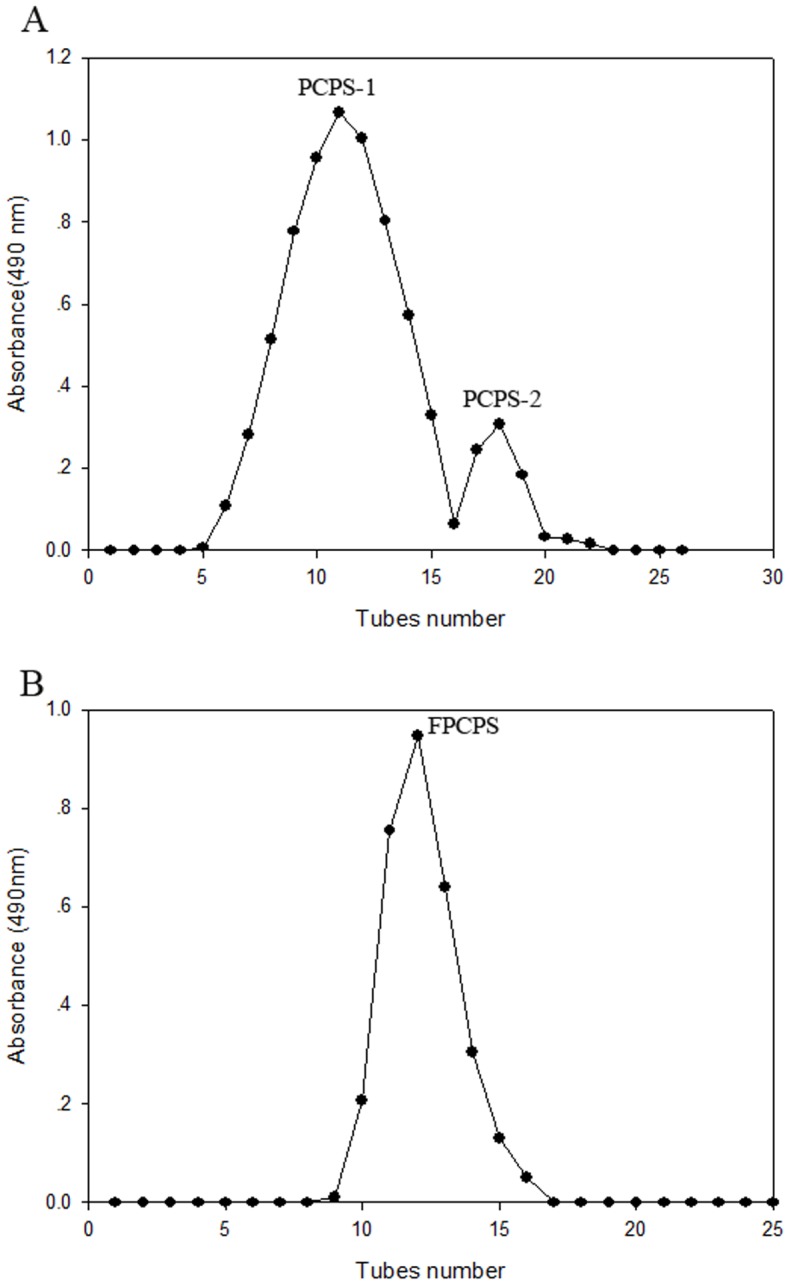
Elution profiles of the crude PCPS from *P. cicadae* by DEAE-52 column (A) and Sephadex G-200 column (B).

**Figure 3:**
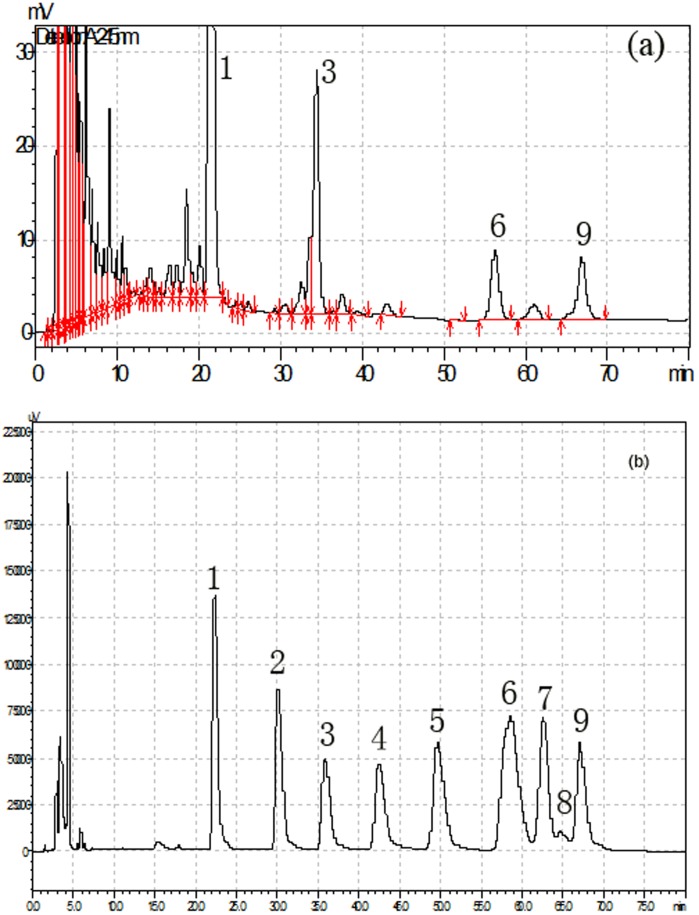
HPLC chromatograms of PMP derivatives of component monosaccharides released from (a) FPCPS and (b) sugar standards. Peaks: 1.Mannose; 2.Ribose; 3.Rhamnose; 4.Glucuronic acid; 5.Glucose; 6.Xylose; 7.Galactose; 8. Fucose; 9.Arabinose.

The crude PCPS was separated and fractionated by DEAE-52 column with gradient elution to give two elution peaks: PCPS-1(91.5%) and PCPS-2 (8.5%), as detected by the phenol-sulfuric acid assay (shown in Figure 2A). Then the main fraction of PCPS-1 was further purified on Sephadex G200 column to present a major peak with a purity of >98.4% in PCPS-1, namely FPCPS (shown in Figure 2B). 2.3% protein was contained in FPCPS, which was estimated by Lowry method. The composition of FPCPS shown in Figure 3, determined by HPLC analysis as PMP derivatives, indicating that it was composed of mannose, rhamnose, xylose and arabinose in a ratio of 43.2: 32.1: 14.5: 10.2.

**Figure 4:**
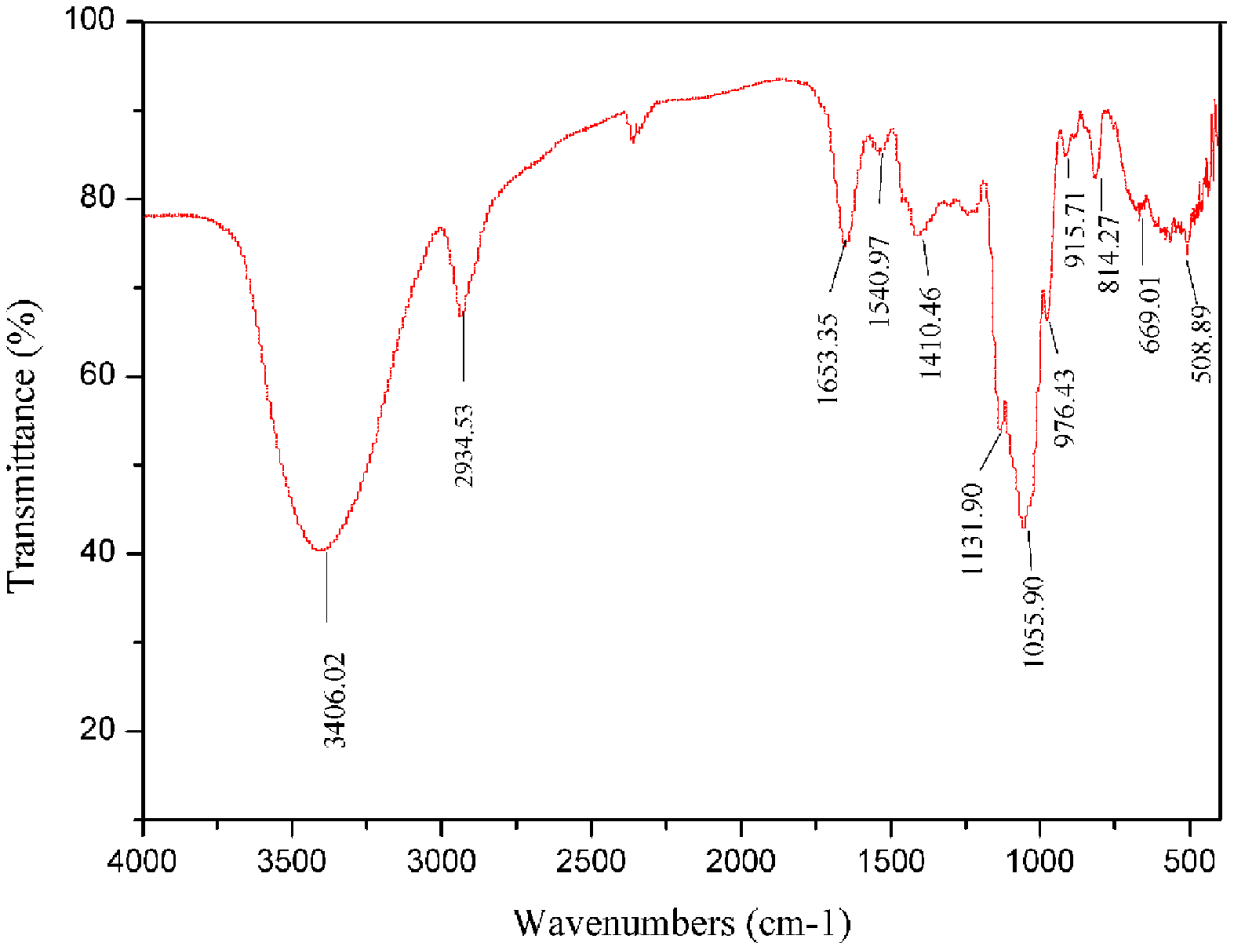
FTIR spectrum of FPCPS produces by *P. cicadae*.

In the IR spectrum of FPCPS (Figure 4), a large absorption peak at 3406 cm^−1^ was OH stretching peak, and C-H stretching was at 2934 cm^−1^. The absorption bands at 1653 cm^−1^ and 1540 cm^−1^ were attributed to the stretching vibration of the carbonyl bond (C = O) of the amide group and the bending vibration of the N-H bond respectively, indicated the existence of protein. Moreover, an obvious characteristic absorption at 814 cm^−1^ was ascribed to the contribution of mannose [28].

**Figure 5:**
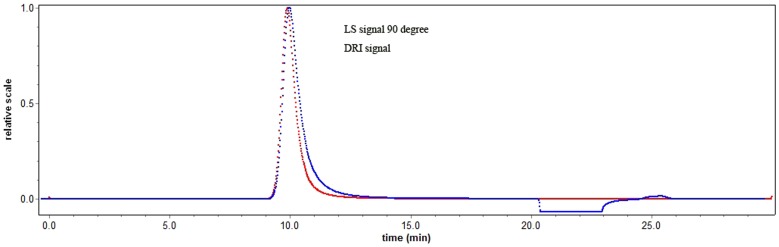
Elution profile of FPCPS for the determination of molecular mass in a SEC-MALLS system.

The SEC/MALLS approach is useful in providing great insight into the characterization of biopolymers without carrying out the elaborate fraction procedures prior to analysis. Recently, SEC instrumentation equipped with both MALLS instrumentation and RI has been used routinely to determine the weight average molecular weight (M_w_) of polymers without the use of standards [29]. Figure 5 shows the elution profile of FPCPS for the determination of molecular mass in a SEC-MALLS system. In this study, the number average molecular weight (M_n_), weight average molecular weight (M_w_) and z-average molecular weights (M_z_) were determined to be 3.153×10^6^ g/mol, 3.754×10^6^ g/mol and 4.594×10^6^ g/mol, respectively. However, the values obtained here were much larger than those reported molecular weights of polysaccharides made by submerged fermentation [30], in which PEPS with only 167 kDa could be achieved from the strain of *Paecilomyces cicadae*. The Rg value was usually a measure regarding how far from the center of mass and how the mass of the polymer chains was concentrated. The (Rg^2^)_z_^1/2^ value for FPCPS was 41.1 nm, which reflected its polymer chains with more compact conformation. And the 1.191 of polydispersity (M_w_/M_n_) indicated that FPCPS has a relatively narrow molecular-weight distribution.

**Figure 6:**
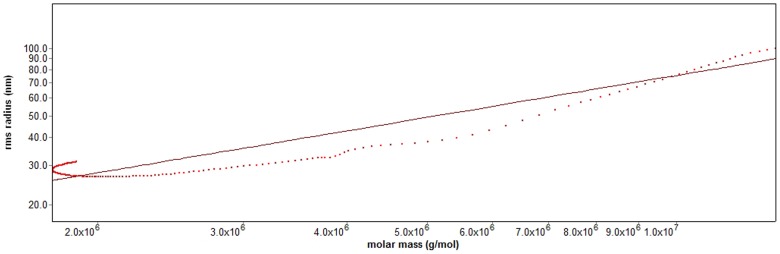
Conformation plot of log (Rg^2^)_z_^1/2^ versus log M_w_ for FPCPS from *P. cicadae*.

To further confirm the above results, the SEC-MALLS-RI system was used to determine the molecular weight and chain conformation of FPCPS by investigating the fractal dimension (d_f_). The power law function (Rg^2^)_z_^1/2^ = kM_w_^1/d^_f_ of polymer in dilute solution can be estimated from many experimental points in the SEC-MALLS chromatogram. Figure 6 shows the plot of RMS radius versus molar mass for FPCPS determined by using the SEC-MALLS-RI system. The d_f_ of such a plot indicates the conformation of FPCPS in solution. Wyatt [31] mentioned that a slope less than or equal 0.33 indicated a homogeneous spherical molecule, however, for linear molecules with random-coil conformation, the slope is generally in the range of 0.5–0.6 and 1.0 reflect the polymer molecular shape of rigid rod. The water-soluble glucan (AF1) extracted from *Auricularia auricular-judae* was reported based on this theory of polymer solution. The findings indicated that the d_f_ value for AF1 in aqueous solution was calculated to be 0.58 for the experimental points in the M_w_ range from 2.0×10^6^ to 1.8×10^7^. In Figure 6, the resulting conformation plot of FPCPS could be expressed as (Rg^2^)_z_^1/2^ = 0.546 M_w_^0.62±0.02^ (nm) and the d_f_ value of the plot for FPCPS is 0.62, which well indicated it is a Gaussian coil polymer in solution [33]. The results were in good agreement with the conformational parameters of the water-soluble glucan made by Xu et al. [32].

### Antioxidant activities of PCPS

**Figure 7:**
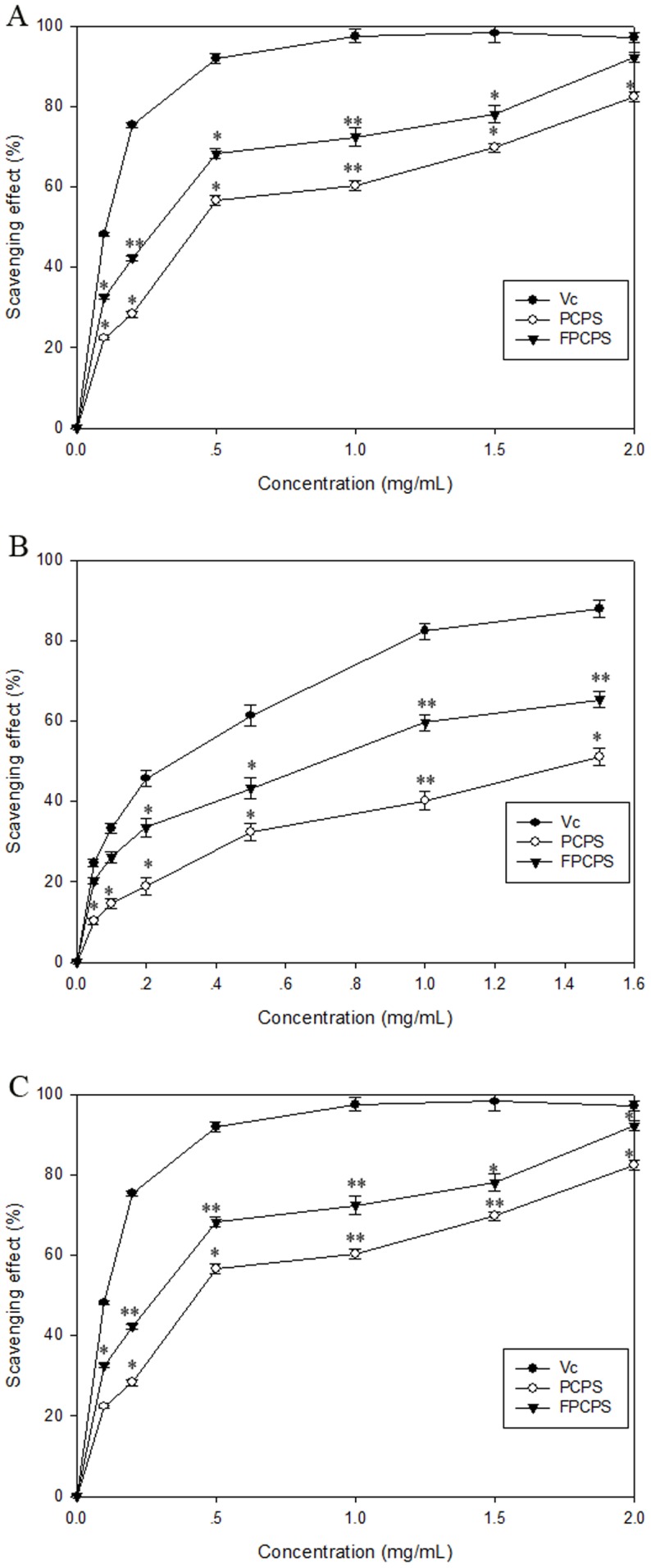
Antioxidant activity of PCPS and FPCPS with comparison to Vc. (A), scavenging effect of PCPS and FPCPS on DPPH radicals compared with that of Vitamin C (standard control); (B), Scavenging effect of PCPS and FPCPS on superoxide radicals compared with that of Vitamin C (standard control); (C), scavenging effect of PCPS and FPCPS on hydroxyl radicals compared with that of Vitamin C (standard control). Results are expressed as mean ± standard deviation of three parallel measurements. Significant differences from control were evaluated using *t*-test; *p<0.05, **p<0.01.

The method of scavenging the stable •DPPH radical is the well accepted way to evaluate the scavenging effect of natural compounds. Figure 7A illustrates that a concentration-dependent, radical-scavenging ability at a series of concentration of Vc, PCPS and FPCPS, the scavenging effects of them were 98.5%, 70.5% and 76.1% at the dose of 1.0 mg/mL respectively. These results suggested that both PCPS and FPCPS have an obvious effect on scavenging free DPPH radical at relatively low amount of addition. From dose of 0.1 to dose of 0.6 mg/mL, FPCPS could donate more hydrogen to combine with DPPH radical when it was purified completely, which was slightly higher than that of polysaccharide produced by submerged culture condition [30].

Superoxide anion radicals are precursors for active free radicals that have potential to react with biological macromolecules and thereby inducing tissue damage. Figure 7B shows approximately identical change trend of scavenging superoxide anion activity among Vc, PCPS, and FPCPS. The scavenging effect of FPCPS was relatively higher than that of PCPS from 0.5 mg/mL to 1.5 mg/mL. The percentage inhibition of superoxide anion generation by 1 mg/mL concentration of PCPS and FPCPS were found to be 40.2% and 59.6% respectively, which could bear comparison with that of Vc [34]. It's noteworthy that FPCPS has a larger molar weight than that of the polymer from the fruiting body and liquid fermentation. Herein, the result of moderate scavenging ability of FPCPS could be well in accordance with the finding of correlation between the antioxidant activity of polysaccharide and its hydroxyl content.

In hydroxyl radical-scavenging assay, the activity of two types of PCPS and Vc used as a positive control were determined. As illustrated in Figure 7C, both PCPS and FPCPS were found to display the ability to scavenge hydroxyl radicals even at relatively low dosage, and then the samples exhibited a slow increase in scavenging effect above 0.5 mg/mL. Our SEC-MALLS data confirmed that the advanced structure of FPCPS was one kind of coil conformation, which might facilitate the formation of intermolecular force in FPCPS and delay the antioxidant activity at relatively high amount. Therefore, the antioxidant mechanism of FPCPS may be attributed to strong hydrogen donating ability and its effectiveness as scavenger of hydroxyl radicals [35]. It is considered that the antioxidant properties of PCPS reported here will provide the experimental evidences for supporting the folkloric uses of *P. cicadae* as a substitute for *C. sinensis*.

## Conclusions

In the present study, the main factors for the solid-state fermentation conditions of *P. cicadae* were scientifically selected and optimized using statistical methods of Plackett-Burman. And the use of the response surface method not only helped in locating the optimum levels of the most significant factors considered with minimum resources and time but also proved to be useful and satisfactory in this process-optimizing practice. Through these optimization experiments, highest yield of PCPS at 10.76 mg/g was obtained when optimum process conditions using *Paecilomyces cicadae* (Miquel) Samson were relative humidity 56.07%, inoculum 13.51 mL/100 g and temperature 27.09°C. The predicted value was well in agreement with the experimental value by validation experiments, which confirmed the availability and the accuracy of the model.

Then FPCPS was obtained by the separation of DEAE-Sepharose FF and Sephacryl S200 chromatography, which was mainly composed of mannose (43.2%), rhamnose (32.1%), xylose (14.5%) and arabinose (10.2%). Based on size exclusion chromatography combined with multi-angle laser light scattering (SEC-MALLS) analysis, FPCPS adopted a Gaussian coil conformation in 0.1 M NaNO_3_ solution with 3.754×10^6^ g/mol of the weight-average molar mass (M_w_) and 41.1 nm of the root-mean square radius (Rg^2^)_z_^1/2^. Furthermore, both PCPS and FPCPS were revealed to show strong antioxidant activities by evaluating in DPPH radical, superoxide radicals and hydroxyl radical assay with comparison to Vc. It is believed that the biopolymers of *Paecilomyces cicadae* (Miquel) Samson produced by solid-state fermentation plays an active function related to its biological activities, and so they should be explored as potential therapeutics.

## References

[1] Bi HT, Gao TT, Li ZH, Ji L, Yang W, et al (2013) Structural elucidation and antioxidant activity of a water-soluble polysaccharide from the fruit bodies of *Bulgaria inquinans* (Fries). Food Chem 138: 1470–1475. 23411269 10.1016/j.foodchem.2012.11.039

[2] Gandhi HP, Ray RM, Patel RM (1997) Exopolymer production by *Bacillus* species. Carbohydr Polym 34: 323–327.

[3] Asker MMS, Ahmed YM, Ramadan MF (2009) Chemical characteristics and antioxidant activity of PCPS fractions from *Microbacterium terregens* . Carbohydr Polym 77: 563–567.

[4] Vinderola G, Perdigón G, Duarte J, Farnworth E, Matar C (2007) Effects of the oral administration of the PCPS produced by *Lactobacillus kefiranofaciens* on the gut mucosal immunity. Cytokine 36: 254–260. 10.1016/j.cyto.2007.01.003 17363262

[5] Yamada T, Ogamo A, Saito T, Watanabe J, Uchiyama H, et al (1997) Preparation and anti-HIV activity of low-molecular weight carrageenans and their sulfated derivatives. Carbohydr Polym 32: 51–55.

[6] Peng Y, Zhang L, Xu X (2003) Characterization of molecular mass of six watersoluble polysaccharide–protein complexes from *Ganoderma tsugae* mycelium. Chin J Polym Sci 21: 309–316.

[7] Kojima T, Tabata K, Itoh W, Yanaki T (1986) Molecular weight dependence of the antitumor activity of schizophyllan. Agric Biol Chem 50: 231–232.

[8] Chen XY, Xu XJ, Zhang LA, Zeng FB (2009) Chain conformation and anti-tumor activities of phosphorylated (1→3)-β-D-glucan from *Poria cocos* . Carbohydr Polym 78: 581–587.

[9] Fukatsu T, Sato H, Kuriyama H (1997) Isolation, inoculation to insect host, and molecular phylogeny of an entomogenous fungus *Paecilomyces tenuipes* . J Invertebr Pathol 70: 203–208. 9367727 10.1006/jipa.1997.4696

[10] Lin RS, Liu HH, Wu SQ, Pang LF, Jia MS, et al (2012) Production and *in vitro* antioxidant activity of PCPS by a mutant, *Cordyceps militaris* SU5-08. Int J Biol Macromol 51: 153–157. 22542852 10.1016/j.ijbiomac.2012.04.011

[11] Takano F, Yahagi N, Yahagi R, Takada S, Yamaguchi M, et al (2005) The liquid culture filtrates of *Paecilomyces tenuipes* (Peck) Samson ( = *Isaria japonica Yasuda*) and *Paecilomyces cicadae* (Miquel) Samson ( = *Isaria sinclairii* (Berk.) Llond) regulate Th1 and Th2 cytokine response in murine Peyer's patch cells *in vitro* and *ex vivo* . Int Immunopharmacol 5: 903–916. 15778126 10.1016/j.intimp.2005.01.005

[12] Cheng JW, Wang YB, He L, Qian H, Fu LZ, et al (2012) Optimization of fermentation process for the production of intracellular polysaccharide from *Paecilomyces cicadae* and the immuno-stimulating activity of intracellular polysaccharide. World J Microb Biot 28: 3293–3299. 10.1007/s11274-012-1140-0 22864602

[13] Balusu R, Paduru RR, Kuravi S, Seenayya G, Reddy G (2005) Optimization of critical medium components using response surface methodology for ethanol production from cellulosic biomass by *Clostridium thermocellum* SS19. Process Biochem 40: 3025–3030.

[14] He L, Xu YQ, Zhang XH (2008) Medium factor optimization and fermentation kinetics for phenazine-1-carboxylic acid production by *Pseudomonas* sp. M18G. Biotechnol Bioeng 100: 250–259. 18078294 10.1002/bit.21767

[15] Liu RS, Li DS, Li HM, Tang YJ (2008) Response surface modeling the significance of nitrogen source on the cell growth and *Tuber* polysaccharides production by submerged cultivation of Chinese truffle *Tuber sinense* . Process Biochem 43: 868–876.

[16] Dubois M, Gilles KA, Hamilton JK, Rebers PA, Smith F (1956) Colorimetric method for determination of sugars and related substances. Anal Chem 28: 350–356.

[17] Plackett RL, Burman JP (1946) The design of optimum multifactorial experiments. Biometrika 33: 305–325.

[18] Papagianni M (2004) Fungal morphology and metabolite production in submerged mycelial processes. Biotechnol Adv 22: 189–259. 14665401 10.1016/j.biotechadv.2003.09.005

[19] Gao H, Liu M, Liu JT, Dai HQ, Zhou XL, et al (2009) Medium optimization for the production of avermectin B1a by *Streptomyces avermitilis* 14-12A using response surface methodology. Bioresour Techol 100: 4012–4016. 10.1016/j.biortech.2009.03.013 19356927

[20] Li Q, Li YM, Han S, Liu YZ, Song DX, et al (2013) Optimization of fermentation conditions and properties of an exopolysaccharide from *Klebsiella* sp. H-207 and application in adsorption of hexavalent chromium. PloS One 8: 1–11. 10.1371/journal.pone.0053542 PMC3539975 23320092

[21] Lowry OH, Rosenbrough NJ, Farr AL, Randall RJ (1951) Protein measurement with the Folin phenol reagent. J Biol Chem 193: 265–275. 14907713

[22] Lv Y, Yang XB, Zhao Y, Ruan Y, Yang Y, et al (2009) Separation and quantification of component monosaccharides of the tea polysaccharides from *Gynostemma pentaphyllum* by HPLC with indirect UV detection. Food Chem 112: 742–746.

[23] Han Q, Yu QY, Shi J, Xiong CY, Ling ZJ, et al (2011) Molecular characterization and hypoglycemic activity of a novel water-soluble polysaccharide from tea (*Camellia sinensis*) flower. Carbohydr Polym 86: 797–805.

[24] Blois MS (1958) Antioxidant determinations by the use of a stable free radical. Nature 181: 1199–1200.

[25] Robak J, Gryglewski RJ (1988) Flavonoids are scavengers of superoxide anions. J Ethnopharmacol 23: 345–348. 10.1016/0006-2952(88)90169-4 2830882

[26] He ZS, Luo H, Cao ZH, Cui ZW (2004) Photometric determination of hydroxyl free radical in Fenton system by brilliant green. Am J Chin Med 243: 236–237.

[27] Moraine RA, Rogovin P (1966) Kinetics of polysaccharide B-1459 fermentation. Biotechnol Bioeng 8: 511–524.

[28] Chen Y, Xie MY, Nie SP, Li C, Wang YX (2008) Purification, composition analysis and antioxidant activity of a polysaccharide from the fruiting bodies of Ganoderma atrum. Food Chem 107: 231–241.

[29] Cui J, Goh KK, Archer R, Singh H (2007) Characterisation and bioactivity of protein-bound polysaccharides from submerged-culture fermentation of *Coriolus versicolor* Wr-74 and ATCC-20545 strains. J Ind Microbiol Biotechnol 34: 393–402. 17318488 10.1007/s10295-007-0209-5

[30] He L, Wu XQ, Cheng JW, Li HB, Lu XY (2010) Purification, composition analysis and antioxidant activity of exopolysaccharides from mycelial culture of *Paecilomyces cicadae* (Miq.) Samson (Ascomycetes). Int J Med Mushrooms 12: 51–62.

[31] Wyatt PJ (1993) Light scattering and the absolute characterization of macromolecules. Anal Chim Acta 272: 1–40.

[32] Xu SQ, Xu XJ, Zhang LN (2012) Branching structure and chain conformation of water-soluble glucan extracted from *Auricularia auricular-judae* . J Agric Food Chem 60: 3498–3506. 22404892 10.1021/jf300423z

[33] Mohammadifar MA, Musavi SM, Kiumarsi A, Williams PA (2006) Solution properties of targacanthin (water-soluble part of gum tragacanth exudate from *Astragalus gossypinus*). Int J Biol Macromol 38: 31–39. 16469374 10.1016/j.ijbiomac.2005.12.015

[34] Leung PH, Zhao S, Ho KP, Wu JY (2009) Chemical properties and antioxidant activity of exopolysaccharides from mycelial culture of *Cordyceps sinensis* fungus Cs-HK1. Food Chem 114: 1251–1256.

[35] Kelvin KTG, Lara MM, D. Neil P, Carolina S, Harjinder S (2011) Molecular characteristics of a novel water-soluble polysaccharide from the New Zealand black tree fern (*Cyathea medullaris*). Food Hydrocoll 25: 286–292.

